# Effects of XIAP on high fat diet-induced hepatic steatosis: a mechanism involving NLRP3 inflammasome and oxidative stress

**DOI:** 10.18632/aging.102559

**Published:** 2019-12-16

**Authors:** Shi Zilu, Huang Qian, Wu Haibin, Ge Chenxu, Lou Deshuai, Li Qiang, Hu Linfeng, Tan Jun, Xu Minxuan

**Affiliations:** 1 Department of Nephrology, First Hospital of Quanzhou Affiliated to Fujian Medical College, Quanzhou, Fujian 362000, China; 2 Department of Teaching and Research Section of Physiology, Basic Medicine Department, Quanzhou Medical College, Quanzhou, Fujian 362000, China; 3 Department of Health Management Center, The Fourth Clinical Medical College of Guangzhou University of Chinese Medicine, Shenzhen, Guangdong 518033, China; 4 Research Center of Brain Intellectual Promotion and Development for Children Aged 0-6 Years, Chongqing University of Education, Chongqing 400067, China; 5 Chongqing Key Laboratory of Medicinal Resources in the Three Gorges Reservoir Region, School of Biological and Chemical Engineering, Chongqing University of Education, Chongqing 400067, China

**Keywords:** NAFLD, XIAP, inflammation and oxidative stress, Nrf-2, NLRP3

## Abstract

Increasing evidence indicates that prolonged fat-rich diet (HFD) ingestion is a predisposing factor for metabolic disorder-associated system inflammation and oxidative stress injury, which contributes to the occurrence of non-alcoholic fatty liver disease (NAFLD). NACHT, LRR and PYD domains-containing protein 3 (NLRP3)-mediated inflammatory infiltration was determined to participate in NAFLD. X-linked inhibitor of apoptosis protein (XIAP) was recently confirmed as an essential regulator for apoptosis in cells. However, the role of XIAP in HFD-induced NAFLD is still not understood. Here, XIAP was characterized with respect to HFD-induced NLRP3 inflammasome activation and reactive oxygen species (ROS) generation *in vivo* and palmitate (PA)-treated cells *in vitro*. After HFD administration, hepatic injury was confirmed via histological assessment (grading and staging of NAFLD) and biochemical parameters, oxidative stress, and reduced antioxidant activity. Up-regulated hepatic dysfunction were further indicated by elevated dyslipidemia, lipid accumulation, and decreased fatty acid β-oxidation associated gene expression. Moreover, in the absence of XIAP, NLRP3 signaling activated by HFD-triggered oxidative stress was up-regulated, accompanied by reduction in antioxidants including HO-1, NQO-1, GST, SOD and Nrf2 activity. The detrimental effects of XIAP blocking on hepatic steatosis and related pathologies were also confirmed in PA-treated mouse liver cells. In contrast, overexpression of XIAP by transfection *in vitro* restrained PA-stimulated hepatic steatosis by suppression of oxidative stress, NLRP3 related inflammatory response, and impairment of Nrf2 activity, further alleviating abnormal metabolic disorder associated NAFLD. Taken together, the present study helped to elucidate how HFD-induced hepatic steatosis was regulated by XIAP, possibly via the inhibition of NLRP3 signaling and oxidative stress injury.

## INTRODUCTION

In recent years, both rapid socioeconomic developments and improvements in standards of living have led to an increase in the consumption of fat-rich foods. Several studies have demonstrated that a high-fat diet (HFD) increases the risk of systemic metabolic syndrome, which includes hyperlipidemia, endotoxemia, obesity, hypertension, and cardio-cerebrovascular disease [1–7]. Notably, fat-rich diets are associated with an increased risk of neuroinflammation and chronic kidney and liver inflammation, potentially through the alteration of the intestinal microbial environment [8–10]. Nonalcoholic fatty liver disease (NAFLD) is an acquired, metabolic stress-induced hepatic injury that occurs when lipids over-accumulate in the liver due to excessive nutrient ingestion [11, 12]. Around 10-20% of NAFLD patients develop non-alcoholic steatohepatitis (NASH), which can ultimately lead to serious and irreversible liver damage, such as cirrhosis and liver cancer [11, 12]. NAFLD is considered the liver manifestation of metabolic syndrome, in which insulin resistance, systemic inflammation, oxidative stress, and abnormal lipid metabolism contribute to the progression of the disease [11]. Therefore, controlling the development of NAFLD may help inhibit the progression of severe liver disease and metabolic disorders. Unfortunately, treatment strategies for NAFLD are progressing slowly, likely due to an inadequate understanding of the signaling pathways involved in its pathogenesis [12]. As such, further study of the molecular mechanisms that contribute to the development and progression of NAFLD and related metabolic disorders is essential.

Increasing evidence has suggested that HFD-induced oxidative stress plays a key role in the development of metabolic syndrome-associated systemic inflammation and NAFLD [13–15]. The presence of free fatty acids in the systemic circulation stimulates the generation of reactive oxygen species (ROS), resulting in redox status dysfunction and hepatic inflammation and lipid deposition [16–18]. However, the molecular mechanisms underlying the crosstalk among hepatic inflammation, NAFLD, and oxidative stress has not yet been fully characterized. X-linked inhibitor of apoptosis protein (XIAP), also known as an inhibitor of apoptosis protein 3 (IAP3) and baculoviral IAP repeat-containing protein 4 (BIRC4), is an important member of the inhibitor of apoptosis protein (IAP) family. XIAP plays a vital role in the regulation of apoptosis by inhibiting caspase-8/receptor-interacting protein kinase 3 (RIPK3), which diminishes both the inflammasome activity of NACHT, LRR, and PYD domains-containing protein 3 (NLRP3) and the generation of pro-inflammatory cytokines [19–21]. Previous studies have indicated that XIAP dysfunction may increase cellular sensitivity to oxidative stress [20, 22]. Regulation of XIAP in anti-apoptosis is closely associated with the activation of nuclear factor (erythroid-derived 2)-like 2 (Nrf2) [23]. Loss of XIAP function may contribute to stimulant-triggered inflammatory responses and apoptosis, as well as to increases in oxidative stress, which, in turn, suppress Nrf2-mediated anti-oxidative activity [22, 24]. However, the role that XIAP plays in HFD-induced NAFLD and the crosstalk between oxidative stress and Nrf2 activation remains unclear. Clarification of how XIAP protects against HFD-stimulated NAFLD, either through direct or indirect interaction with anti-oxidative stress proteins, is needed.

In this study, an XIAP knockdown mouse model was established by transfection with ItsiRNA to evaluate the influence of a prolonged fat-rich diet on XIAP-associated inflammatory response and oxidative stress *in vivo*. To determine the effects of both XIAP knockdown and overexpression *in vitro*, hepatic cell lines were treated with palmitate (PA) to model hepatic fat accumulation.

## RESULTS

### XIAP is down-regulated in the livers of both HFD-fed mice and palmitate-treated cultured hepatocytes

**Figure 1:**
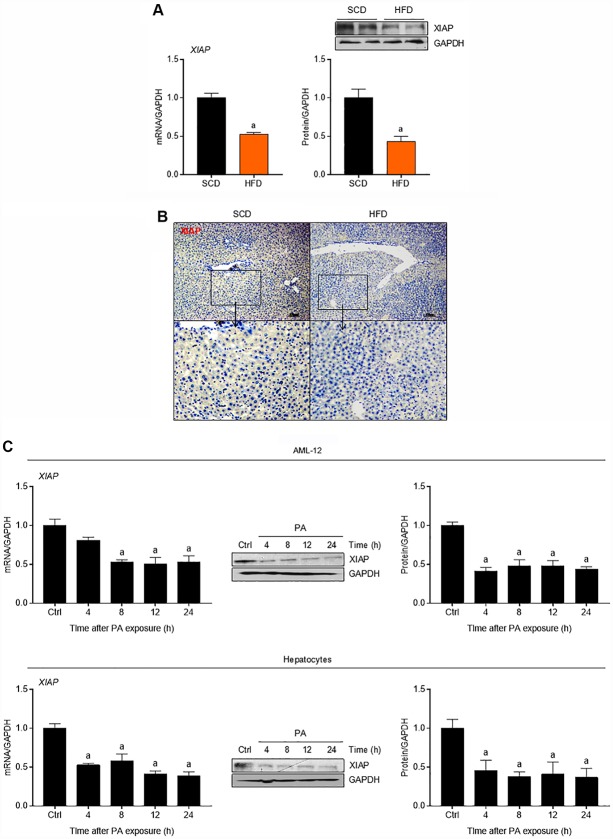
**XIAP is down-regulated in fatty liver of mice fed with HFD.** (**A**) qPCR, immunoblotting and (**B**) immunohistochemical analysis of XIAP expression in liver tissues from scrambled RNA mice fed a SCD or HFD for 12 weeks. (**C**) Primary mice hepatocytes and normal AML-12 cells were treated with 250 μM palmitate (PA) for 0, 4, 8, 12 or 24 hours, followed by determination of XIAP expression in mRNA and protein levels using qPCR or western blotting detection. For all bar plots shown, data are expressed as the mean ± SEM. n = 8 per group. ^a^p < 0.05 vs. SCD or control group (Ctrl).

To determine whether fat accumulation in the liver affects XIAP expression *in vivo*, mice were fed either an HFD or a standard chow diet (SCD) as a control. As shown in Figure 1A, HFD mouse livers had significantly lower expression levels of both XIAP mRNA and protein compared to the controls. Immunohistochemical (IHC) assay further demonstrated the down-regulation of XIAP in HFD-fed mouse livers (Figure 1B). To determine whether fat accumulation in the liver affects XIAP expression *in vitro*, both primary hepatocytes and AML-12 cells were incubated with 250 μM PA for 0, 4, 8, 12, or 24 h. As expected, both XIAP mRNA and protein expression levels were significantly decreased in the PA-treated cells compared to controls, and this decrease occurred in a time-dependent manner (Figure 1C). These results suggest that XIAP likely participates in the regulation of HFD-induced fatty liver.

### XIAP knockdown aggravates metabolic syndrome, liver inflammation, and hepatic steatosis in HFD-fed mice

**Figure 2:**
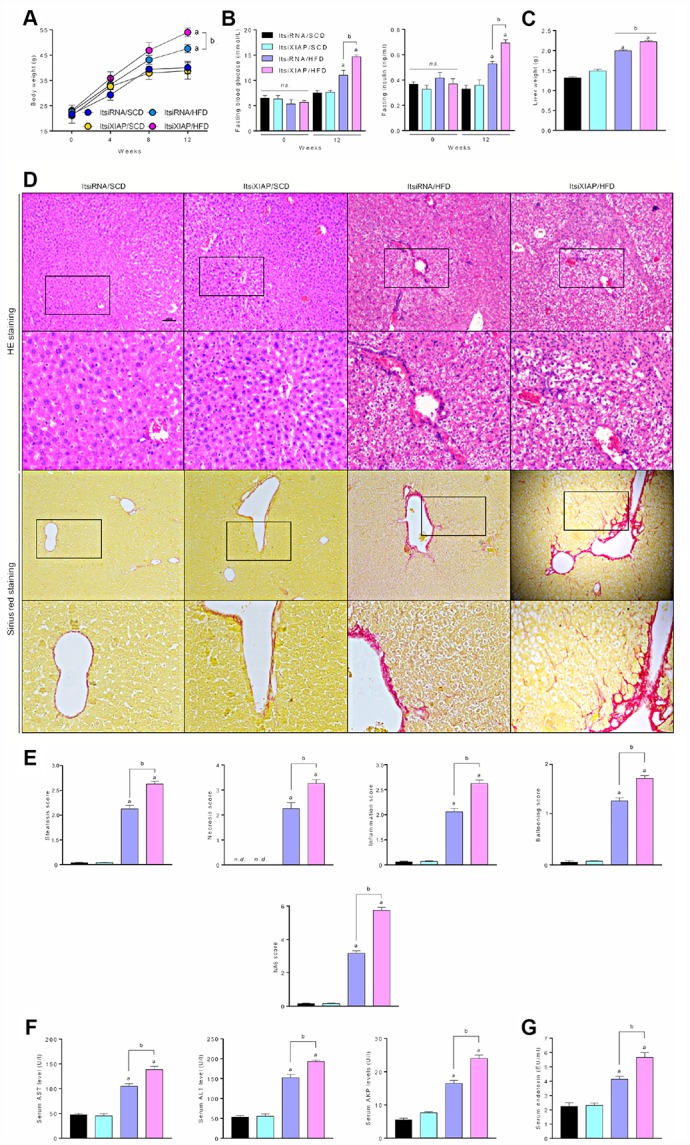
**XIAP blocking promotes metabolic syndrome and hepatic steatosis in HFD-fed mice.** (**A**) Alteration of mice body weight in ItsiRNA or ItsiXIAP, and co treated with prolonged HFD ingestion group was examination every 4 weeks. (**B**) Fasting blood glucose levels (left) and fasting serum insulin levels (right) in mice fed with NCD or HFD for 0 or 12 weeks. (**C**) The ratio of liver weight to body weight was measured. (**D**) Representative hematoxylin-eosin (HE)-stained and sirius red-stained liver sections. (**E**) The steatosis, necrosis, inflammation, ballooning and NAS score were quantified. (**F**) Serum AST, ALT, AKP and (**G**) Serum endotoxin at 12 weeks were measured. For all bar plots shown, data are expressed as the mean ± SEM. n = 8 per group. ^a^p < 0.05 vs. ItsiRNA/SCD or ItsiXIAP/SCD. ^b^p < 0.05 vs. ItsiRNA/HFD.*n.s*., no significant difference.

In order to elucidate the role of XIAP in hepatic steatosis and its complications, XIAP knockdown mice were generated by *in vivo* siRNA transfection and then fed an HFD for 12 weeks. Prior to this, the efficiency of XIAP siRNA *in vivo* in high fat diet induced fatty liver were confirmed using qPCR and western blotting assay at 1^st^, 4^th^, 8^th^, and 12^th^ week during the period of *in vivo* siRNA transfection, as indicated in Supplementary Figure 1. Accordingly, as shown in Figure 2A, the recorded body weights at 0, 4, 8, and 12 weeks were significantly increased in both ItsiRNA/HFD and ItsiXIAP/HFD mice compared to SCD-fed mice, suggesting that a long-term HFD leads to dramatic weight fluctuations in mice. In addition, knockdown of XIAP *in vivo* may result in a further increase in mouse body weight, as previous studies have demonstrated that a prolonged HFD significantly contributed to the development of metabolic disorder [3, 10].

To identify the effect of XIAP on HFD-induced systemic metabolic syndrome and hepatic inflammation, fasting blood glucose and insulin levels in 0- and 12-week-old mice were measured. As illustrated in Figure 2B, HFD-fed mice had significantly increased fasting blood glucose and insulin levels compared to controls. Interestingly, ItsiXIAP/HFD mice had higher blood glucose and insulin levels than did the ItsiRNA/HFD group, indicating that XIAP knockdown may have promoted the development of HFD-induced metabolic imbalance in mice. This result is supported by the up-regulation of liver injury-associated indicators in these mice. As shown in Figure 2C, there was a significant increase in the liver wet weight in HFD-fed mice compared to SCD-fed mice, which was due in part to excessive lipid deposition. Moreover, there was a remarkable difference in liver weight between the ItsiXIAP/HFD and ItsiRNA/HFD groups, suggesting that XIAP knockdown itself was capable of altering the weight of livers in HFD-fed mice. To determine the degree of hepatic injury in both the HFD- and SCD-fed mice, livers from these mice were subjected to Hematoxylin and Eosin (H&E) staining and Sirius Red staining (Figure 2D). Higher levels of steatosis, necrosis, and inflammation, as well as higher ballooning and NAFLD activity (NAS) scores (Figure 2E), were observed in ItsiXIAP/HFD and ItsiRNA/HFD mice compared to SCD-fed mice, suggesting that long-term HFD ingestion contributed to the development of inflammatory infiltration in liver and hepatic steatosis. Notably, mice injected with XIAP-siRNA exhibited more serious pathological changes and had a higher risk of developing NAFLD than did mice injected with ItsiRNA/HFD. This indicates that XIAP expression may help to stabilize and recover metabolic balance in HFD-induced fatty livers. Furthermore, mice fed an HFD had increased AST, ALT, and AKP levels and serum endotoxin content, suggesting that an HFD promoted hepatic steatosis in these mice and may be associated with differential regulation of XIAP.

**Figure 3:**
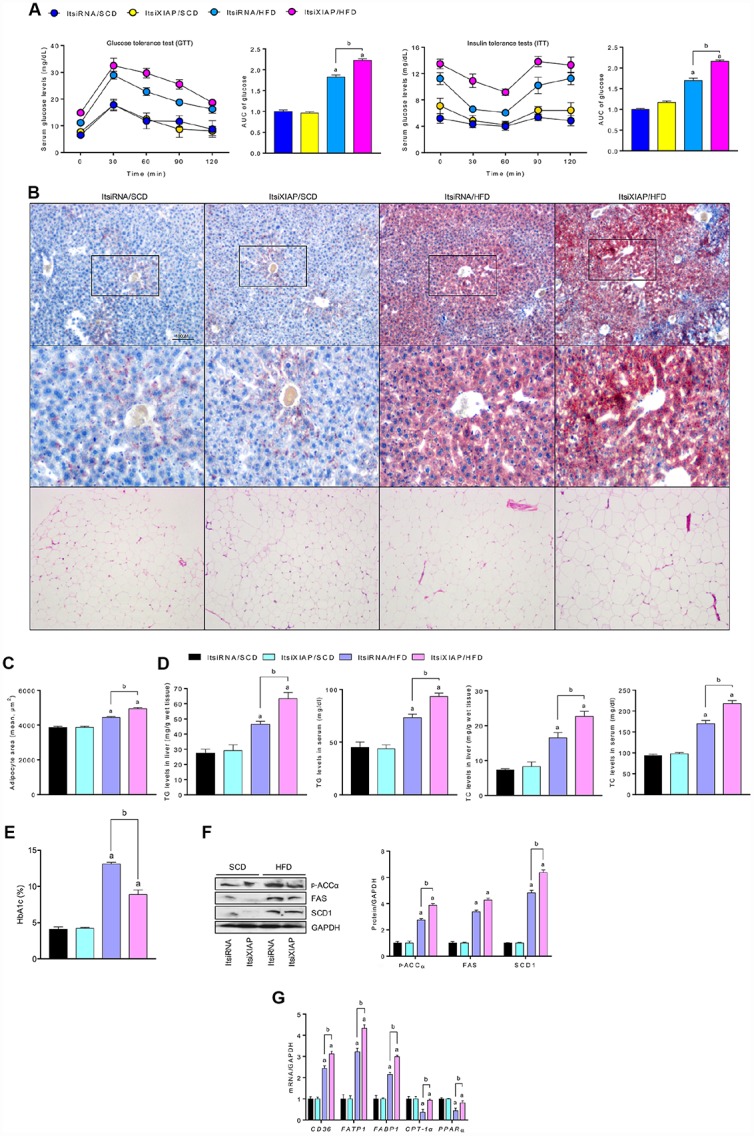
**XIAP knockdown aggravates HFD-induced hepatic dysfunction.** (**A**) Glucose tolerance test (GTT) with area under curve (AUC) (Left), and insulin tolerance tests (ITT) with area under curve (AUC) (Right) in mice fed with SCD or HFD were measured. (**B**) Representative Oil-Red-O-stained images of liver sections (top and medium) and HE-stained adipose tissue (bottom) from each group of mice after SCD or HFD treatment at 12 weeks. (**C**) The quantification of adipocyte area. (**D**) Serum lipid (including TG and TC), liver lipid (including TG and TC) levels and glycosylated hemoglobin (**E**) were assessed. (**F**) Representative western blot analysis for the expression of p-ACCα, FAS and SCD1 in the livers from each group of mice. (**G**) qPCR analysis for genes involved in fatty acid metabolism (CD36, FATP1, FABP1, CPT-1α and PPARα) in the livers of mice were performed. For all bar plots shown, data are expressed as the mean ± SEM. n = 8 per group. ^a^p < 0.05 vs. ItsiRNA/SCD or ItsiXIAP/SCD. ^b^p < 0.05 vs. ItsiRNA/HFD.

To determine whether XIAP activity is essential for the regulation of metabolic balance, glucose tolerance tests (GTT) and insulin tolerance tests (ITT) were used to measure hepatic lipid metabolism indicators in both HFD- and SCD-fed mice As indicated in Figure 3A, ItsiXIAP/HFD and ItsiRNA/HFD mice had higher levels of glucose tolerance and insulin resistance than did the SCD-fed control group. Notably, siXIAP-treated mice exhibited higher glucose levels and dramatic shifts in area under curve (AUC) scores, suggesting that knockdown of XIAP *in vivo* may impair glucose tolerance and insulin sensitivity, further exacerbating metabolic disorders. This hypothesis was supported by the presence of lipid deposition in the liver as measured by oil-red staining (Figure 3B), as well as changes in adipocyte area (Figure 3C), triglyceride (TG) and total cholesterol (TC) levels in the liver or serum (Figure 3D), and the alteration of glycosylated hemoglobin (HbA1c) (Figure 3E). In addition, XIAP knockdown resulted in a significant up-regulation in the expression of genes associated with fatty acid synthesis and uptake (p-ACCα, FAS, SCD1, CD36, FATP1, and FABP1) and a significant down-regulation in the expression of genes related to fatty acid β-oxidation (CPT-1α and PPAR-α) in the livers of mice fed an HFD (Figure 3F and 3G). These data suggest that XIAP plays a vital role in the regulation of HFD-stimulated metabolic imbalance.

**Figure 4:**
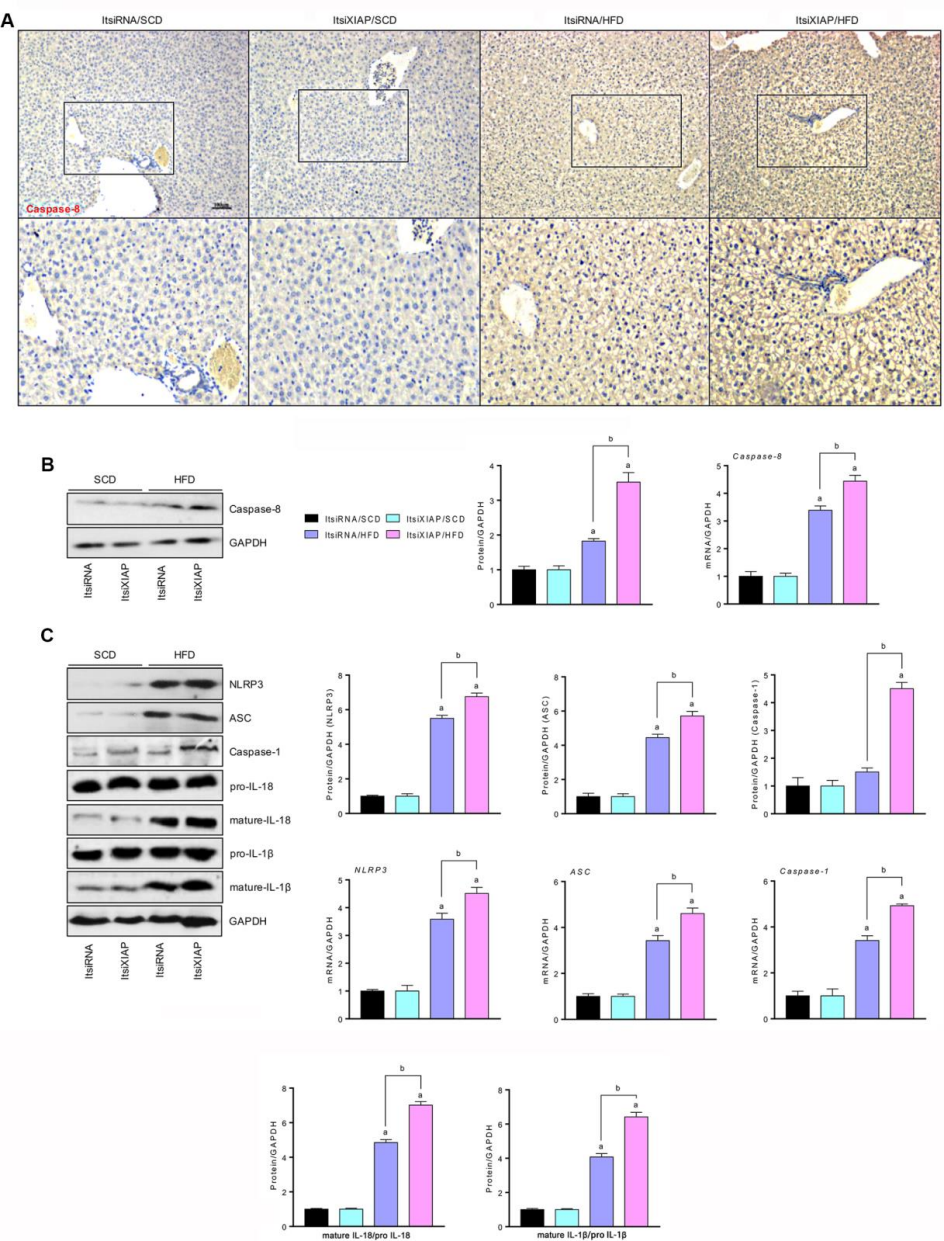
**Impairment of XIAP enhances HFD-hepatic inflammation by up regulation of NLRP3 activity.** (**A**) Representative images of immunohistochemical analysis for Caspase-8 in liver tissue sections. (**B**) Representative western blot analysis (left) and quantification (right) of the expression of Caspase-8 in liver tissues. (**C**) Representative western blot analysis (left) and quantification (right) of the expression of NLRP3 inflammasome (NLRP3, ASC, and Caspase-1) in liver tissues, and bands for pro-IL-18, mature-IL-18, pro-IL-1β and mature-IL-1β. For all bar plots shown, data are expressed as the mean ± SEM. n = 8 per group. ^a^p < 0.05 vs. ItsiRNA/SCD or ItsiXIAP/SCD. ^b^p < 0.05 vs. ItsiRNA/HFD.

Previous studies have shown that XIAP activation was able to suppress the caspase-8/NLRP3 signaling-triggered inflammation response, which was induced significantly by prolonged stimulation [19, 20]. To determine whether NLRP3 expression was altered and how XIAP activity affects NLRP3-mediated inflammatory responses in HFD-induced NAFLD, the expression levels of involved proteins were measured. As shown in Figure 4A and Table 1, IHC detection of caspase-8 in the liver sections showed a significant increase in caspase-8 expression in HFD-treated mice compared to the SCD group. This indicated that the up-regulation of caspase-8 expression contributed to the development of NAFLD in HFD-fed mice. Immunoblotting analysis revealed that NLRP3, mature IL-18, and mature IL-1β were more highly expressed in the fatty livers of HFD-fed mice as compared to the SCD-fed controls (Figure 4B and 4C). Moreover, the mRNA expression levels of NLRP3, ASC, and caspase-1 were increased in HFD-fed mice compared to controls. These results indicate that the activation of NLRP3 signaling is involved in the development of NAFLD

**Table 1:** The immunohistochemical detection score in ItsiRNA/HFD or ItsiXIAP/HFD group.

**Items**	**ItsiRNA/HFD**	**ItsiXIAP/HFD**	***p* value**
Caspase-8	6.43 ± 0.36	8.33 ± 0.33^b^	*p*=0.003
Nrf2	4.94 ± 0.24	3.25 ± 0.26^b^	*p*=0.006
HO-1	4.42 ± 0.21	3.45 ± 0.29^b^	*p*=0.045
SOD1	3.37 ± 0.23	2.16 ± 0.31^b^	*p*=0.029

Data are expressed as the mean ± SEM. ^b^p < 0.05 vs. ItsiRNA/HFD.

### XIAP knockdown exacerbates HFD-induced oxidative stress in fatty livers

**Figure 5:**
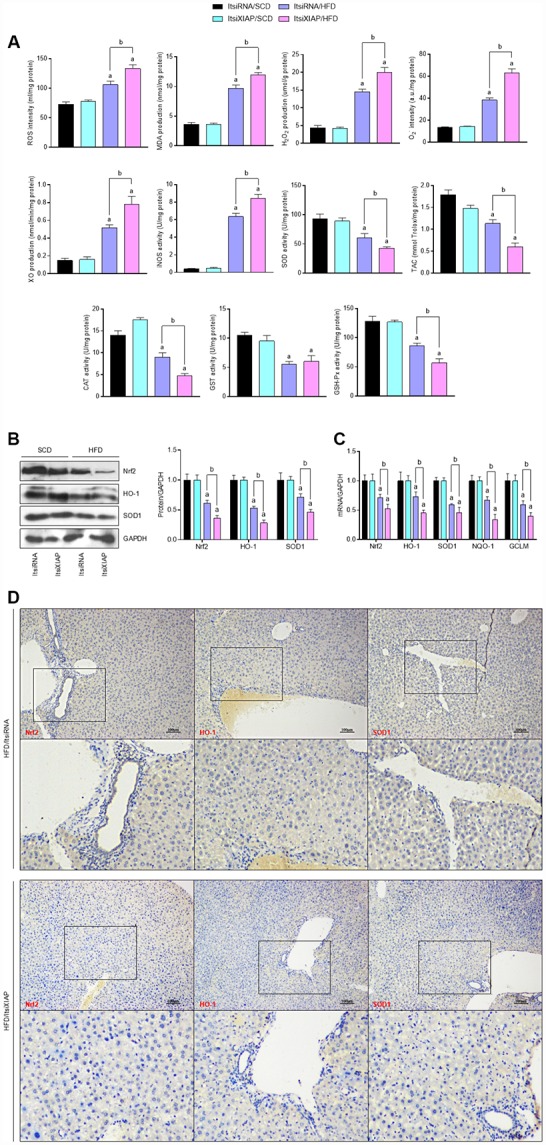
**Knockdown of XIAP promotes HFD-induced oxidative stress in liver mice.** (**A**) Calculation of ROS intensity, MDA, H_2_O_2_, O_2_^-^, XO, iNOS levels, and SOD, TAC, CAT, GST and GSH-Px activity in hepatic tissue samples from each group of mice. (**B** and **C**) Representative western blot analysis of the expression of SOD1, HO-1, Nrf2, and quantification of SOD1, HO-1, Nrf2, NQO-1 and GCLM in liver samples. (**D**) Representative images of immunohistochemical analysis for Nrf2, HO-1 and SOD1 expression in liver tissue sections. For all bar plots shown, data are expressed as the mean ± SEM. n = 8 per group. ^a^p < 0.05 vs. ItsiRNA/SCD or ItsiXIAP/SCD. ^b^p < 0.05 vs. ItsiRNA/HFD.

Previous studies have shown that the long-term intake of excess fat associated with an HFD contributes to the development of obesity, diabetes, cardio-cerebrovascular disease, and nonalcoholic steatohepatitis through the up-regulation of oxidative stress levels [1–7]. In order to better understand the role that XIAP plays in the HFD-induced development of fatty liver, the level oxidative stress in the liver was measured. As expected, the HFD-induced increase in oxidative stress levels was further elevated in the livers of mice with XIAP knockdown. There was an increase in ROS generation, malondialdehyde (MDA) production, H_2_O_2_ and O_2_^-^ content, and XO and iNOS activity, as well as a reduction in SOD, TAC, CAT, GST, and GSH-Px activity in ItsiXIAP/HFD mice compared to ItsiRNA/HFD and ItsiRNA/SCD mice (Figure 5A). Notably, the impairment of hepatic Nrf2 signaling caused by prolonged HFD ingestion was further aggravated in XIAP knockdown mice. This was accompanied by the functional loss of Nrf2, SOD1, NQO-1, HO-1, and GCLM activities in these mice compared to the corresponding control groups (Figure 5B and C). These data were also supported by IHC analysis, which showed that the protein expression levels of Nrf2, HO-1, and SOD1 expression were down-regulated in ItsiXIAP/HFD mice compared to ItsiRNA/HFD mice (Table 1). These results demonstrate that XIAP knockdown exacerbates the effects of HFD-induced NAFLD by triggering an increase in oxidative stress (Figure 5D).

### XIAP knockdown promotes PA-induced lipometabolic disturbance *in vitro*

**Figure 6:**
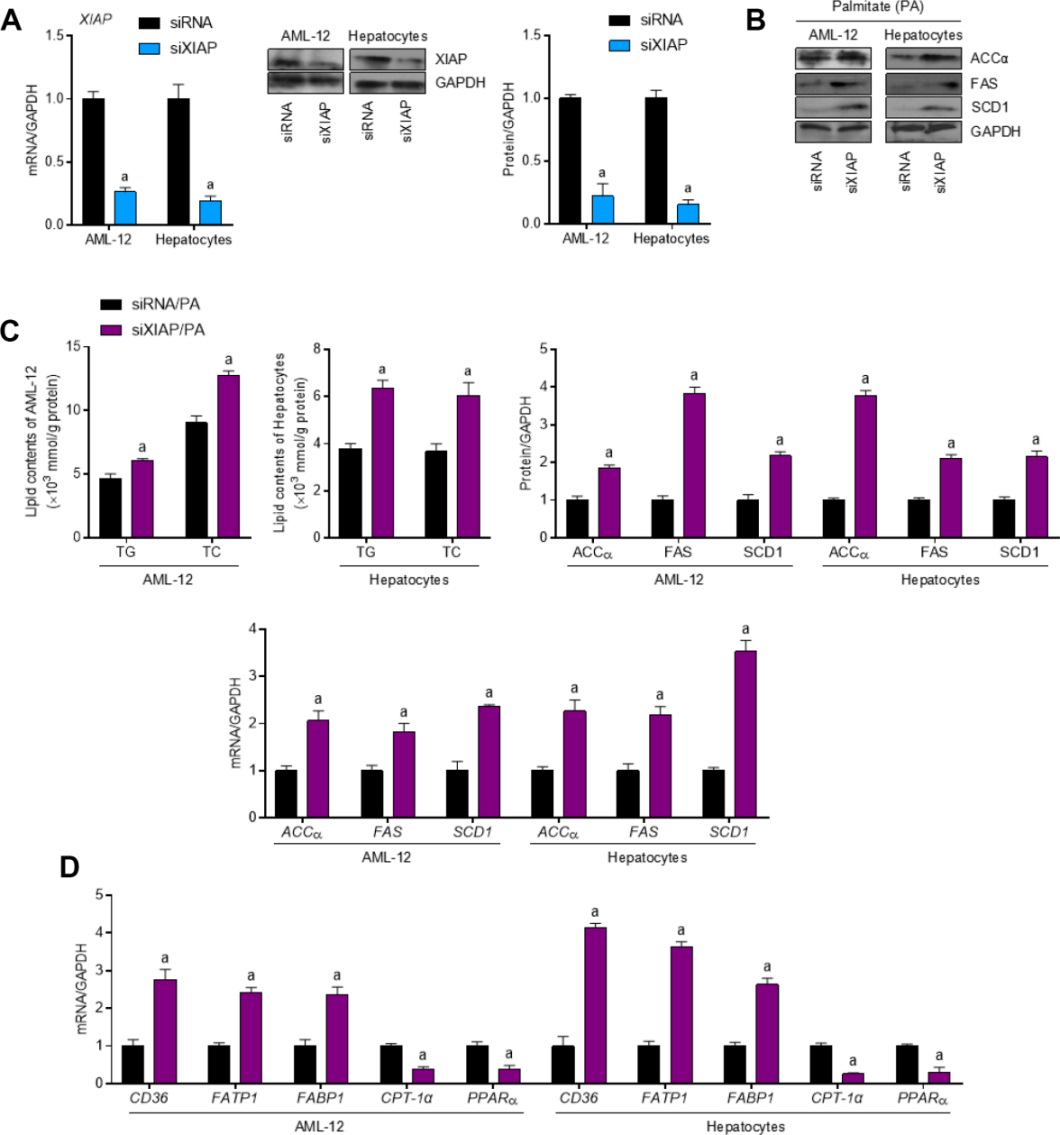
**XIAP silence enhances dyslipidemia in PA-treated cells *in vitro*.** (**A**) AML-12 and primary hepatocytes were transfected with XIAP specific siRNA sequence for 24 h, followed by transfection efficacy calculation using qPCR and western blot analysis. AML-12 and primary hepatocytes were subjected to XIAP siRNA transfection for 24 h, and then cells were incubated with 250 μM of PA for additional 24 h, followed by further investigation. (**B**) Representative western blot and qPCR analysis of the expression of ACCα, FAS and SCD1 in PA-induced hepatocytes and AML-12 cell line. (**C**) Determination of cellular TG and TC levels in AML-12 and hepatocytes was next detected. (**D**) qPCR analysis for genes associated with fatty acid metabolism, including CD36, FATP1, FABP1, CPT-1α and PPARα, in cells treated as indicated. For all bar plots shown, data are expressed as the mean ± SEM. n = 8 per group. ^a^p < 0.05 vs. siXIAP or siRNA/PA.

**Figure 7:**
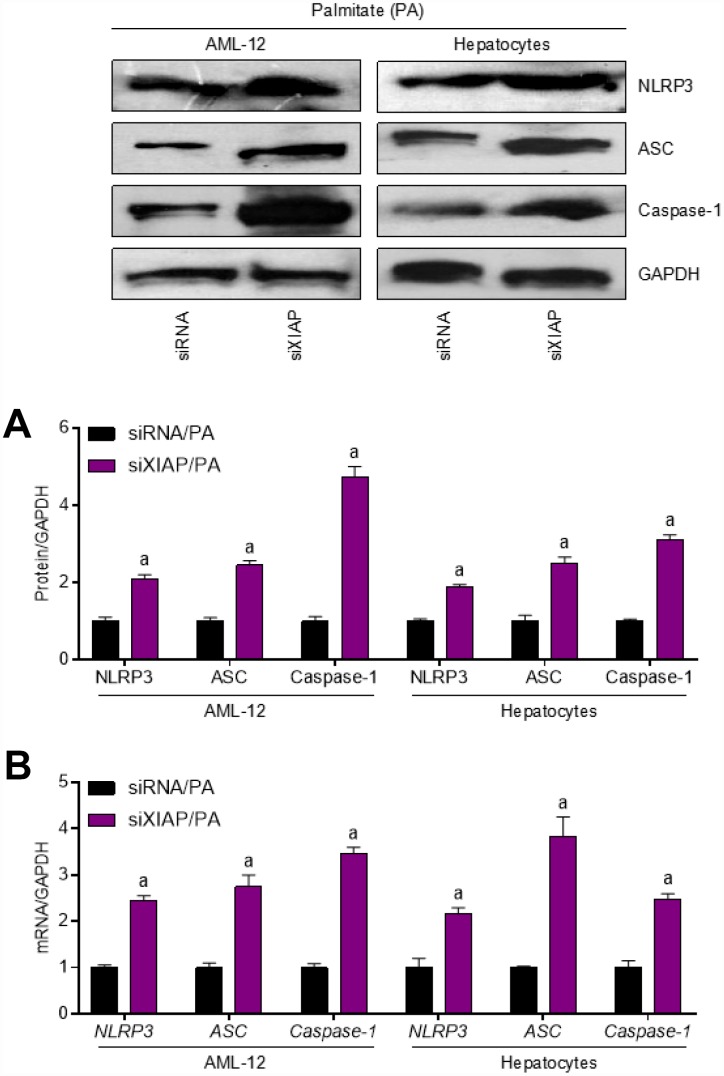
**Inhibition of XIAP positively regulates PA-triggered inflammation *in vitro*.** AML-12 and primary hepatocytes were transfected with XIAP siRNA for 24 h, followed by PA (250 μM) treatment for another 24 h. Then, further studies as exhibited were performed. (**A** and **B**) Representative western blot and qPCR detection showed the changes in mRNA and protein levels of NLRP3 inflammasome (NLRP3, ASC and Caspase-1) in PA-induced cells. For all bar plots shown, data are expressed as the mean ± SEM. n = 8 per group. ^a^p < 0.05 vs. siRNA/PA.

To further define the role of XIAP in HFD-induced hepatic steatosis, primary hepatocytes and AML-12 cells were used to evaluate the differential expression of oxidative stress indicators upon treatment with PA *in vitro*. Both AML-12 cells and primary hepatocytes were transfected with either XIAP siRNA or the corresponding control vectors and then treated with 250 μM PA for 24 h to establish the *in vitro* model. As shown in Figure 6A, XIAP expression was downregulated in both PA-treated AML-12 cells and hepatocytes, indicating that XIAP knockdown efficiency was good. Similar to the *in vivo* results, XIAP knockdown resulted in a significant upregulation of fatty acid synthesis and uptake indicators, including ACCα, FAS, and SCD1, as well as a sharp increase in lipid deposition in PA-treated AML-12 cells and hepatocytes (Figure 6B and 6C). Moreover, changes in fatty acid β-oxidation indicators, such as CPT-1α, PPARα, CD36, FATP1, and FABP1, indicated that they may play a role in the development of lipometabolic disturbances in PA-treated cells. These results suggested that PA treatment could alter lipid homeostasis in liver-related cell lines, promote fatty acid synthesis and uptake, and suppress fatty acid β-oxidation, ultimately increasing lipid accumulation in cells. Additionally, activation of NLRP3 signaling was observed in PA-treated AML-12 cells and hepatocytes. As shown in Figure 7A and 7B, there were significant increases in both NLRP3 mRNA and protein levels, indicating an increase in the activation of NLRP3 signaling in PA-treated cells. Taken together, these data suggest that NLRP3 signaling triggered inflammation-associated lipometabolic disturbance and induced lipid deposition *in vitro.*

### XIAP knockdown facilitates PA-induced oxidative stress *in vitro*

**Figure 8:**
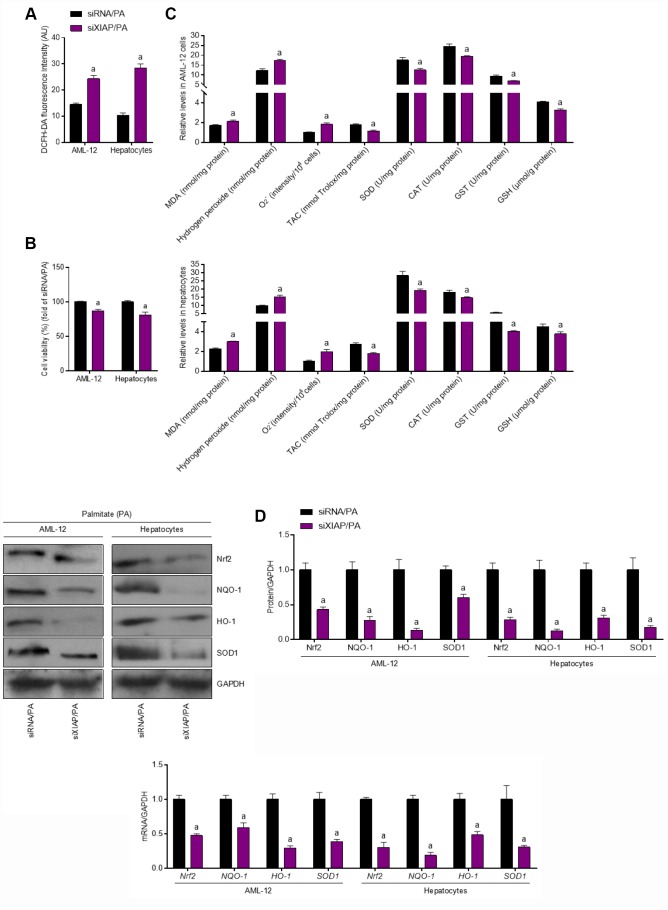
**Functional loss of XIAP contributes to PA-induced oxidative stress *in vitro*.** AML-12 and primary hepatocytes were transfected with XIAP siRNA for 24 h, followed by PA (250 μM) treatment for additional 24 h. Subsequently, further studies were carried out. (**A** and **B**) Determination of cellular ROS production and cell viability. (**C**) Calculation of cellular MDA, H_2_O_2_, O_2_^-^ levels, TAC, SOD, CAT, GST and GSH activities. (**D**) Western blot and qPCR analysis showed the alteration of SOD1, NQO-1, HO-1 and Nrf-2 in cells treated as described. For all bar plots shown, data are expressed as the mean ± SEM. n = 8 per group. ^a^p < 0.05 vs. siRNA/PA.

*In vivo* experiments have shown that XIAP knockdown promoted HFD-induced oxidative stress injury and NLRP3 signaling-related lipid deposition in the liver of mice, further stimulating the development of NAFLD. As a result, the alteration of oxidative stress levels in PA-treated cells was investigated. DCFH-DA analysis revealed that ROS production was increased in PA-treated AML-12 cells and hepatocytes compared to the control groups (Figure 8A), suggesting that PA treatment alone is able to promote the generation of ROS *in vitro*. In addition, 24-h PA treatment reduced the viability of AML-12 cells and hepatocytes (Figure 8B). The functional loss of XIAP in PA-treated cells resulted in both an increase in oxidative stress, as evidenced by an increase in MDA production and H_2_O_2_ and O_2_^-^ content, as well as a decrease in the antioxidant activities ofSOD, TAC, CAT, GST, and GSH (Figure 8C). These observations suggest that PA-induced oxidative stress injury contributes to the imbalance of lipid metabolism in cells. In addition, the activation of Nrf2 signaling was significantly suppressed in PA-treated cells (Figure 8D). Reduction in the mRNA and protein expression of antioxidants, such as NQO-1, HO-1, and SOD1, was more pronounced in these cells compared to the siRNA/PA groups, suggesting that PA promoted oxidative stress injury in cells by downregulating antioxidant production. These results indicate that PA-induced imbalances in cellular lipid metabolism are associated with oxidative stress injury and the suppression of antioxidant production.

### Enhancing XIAP expression reversed PA-induced oxidative stress injury in hepatic cells

**Figure 9:**
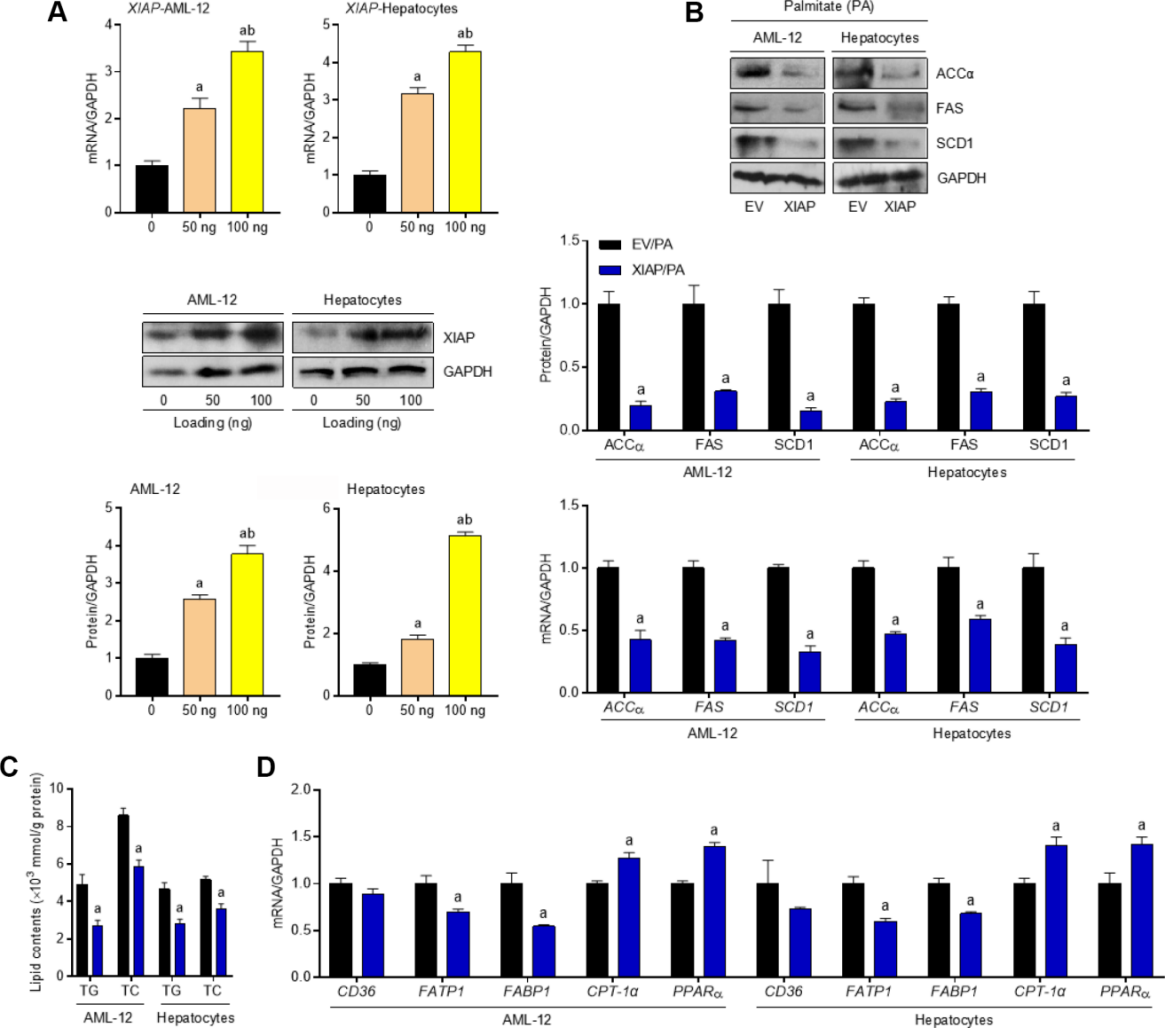
**Activation of XIAP alleviates dyslipidemia in PA-treated cells *in vitro*.** AML-12 and primary hepatocytes were transfected with pUNO1/XIAP plasmid for 24 h, followed by PA (250 μM) treatment for additional 24 h. (**A**) Transfection efficacy calculation using qPCR and western blot detection in 50 ng or 100 ng plasmid-treated cells. (**B**) After transfection with 100 ng vectors, representative western blot and qPCR analysis of the expression of ACCα, FAS and SCD1 in PA-induced hepatocytes and AML-12 cell line. (**C**) Determination of cellular TG and TC levels in AML-12 and hepatocytes was next detected. (**D**) qPCR analysis for genes associated with fatty acid metabolism, including CD36, FATP1, FABP1, CPT-1α and PPARα, in cells treated as indicated. For all bar plots shown, data are expressed as the mean ± SEM. n = 8 per group. ^a^p < 0.05 vs. EV/PA.

**Figure 10:**
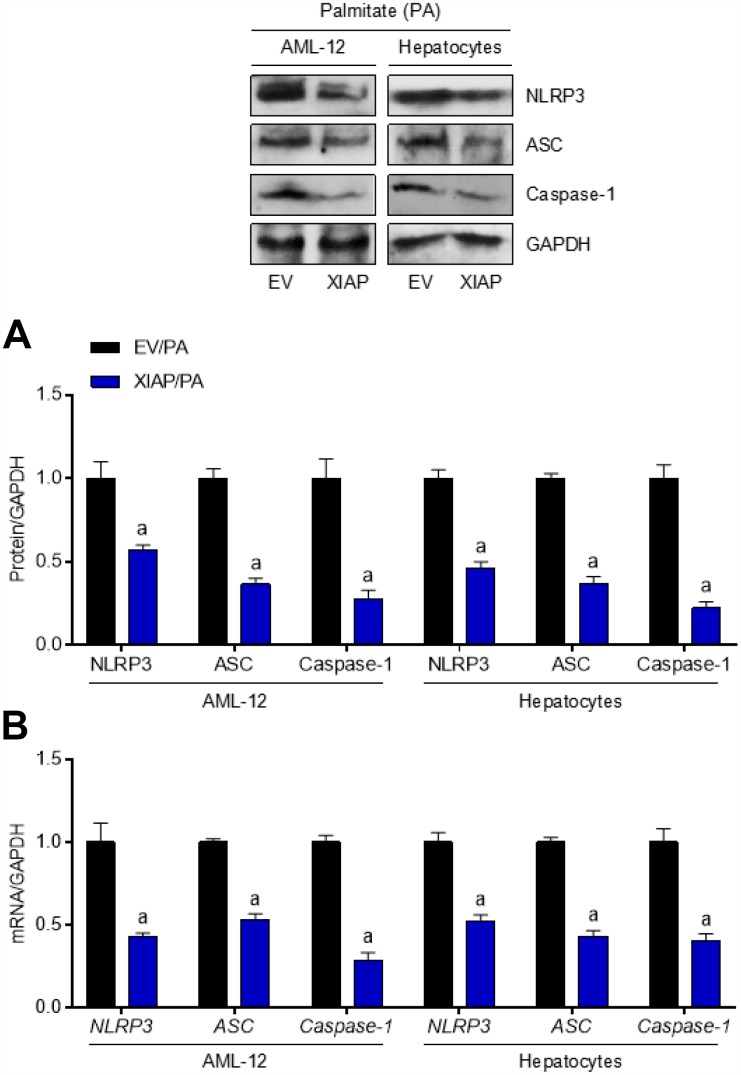
**Up regulation of XIAP levels suppresses PA-triggered inflammation *in vitro*.** AML-12 and primary hepatocytes were transfected with pUNO1/XIAP plasmid for 24 h, followed by PA (250 μM) treatment for additional 24 h. Then, further studies as exhibited were performed. (**A** and **B**) Representative western blot and qPCR detection showed the changes in mRNA and protein levels of NLRP3 inflammasome (NLRP3, ASC and Caspase-1) in PA-induced cells. For all bar plots shown, data are expressed as the mean ± SEM. n = 8 per group. ^a^p < 0.05 vs. EV/PA.

To explain the role that XIAP plays in PA-induced oxidative stress more comprehensively, an XIAP overexpression cell model was constructed. Primary hepatocytes and AML-12 cells were transfected with pUNO1/mXIAP or a corresponding control vector and then incubated with 250 μM PA for 24 h to establish the *in vitro* model. As shown in Figure 9A, hepatocytes and AML-12 cells treated with 50 ng or 100 ng of the vectors exhibited higher expression of both XIAP mRNA and protein compared to the 0 ng loading control group. Both mRNA and protein expression levels of ACCα, FAS, and SCD1 were down-regulated in cells overexpressing XIAP (Figure 9B), indicating that XIAP activation helps maintain lipid metabolism balance in PA-treated cells through the regulation of fatty acid synthesis and uptake genes. Moreover, cells overexpressing XIAP had a decrease in both the lipid content (TC and TG levels) (Figure 9C) and the expression of fatty acid synthesis and uptake genes (ACCα, FAS, SCD1, CD36, FATP1, and FABP1), as well as an up-regulation in the expression of fatty acid β-oxidation genes (CPT-1α and PPAR-α) compared to control cells (Figure 9D). Consistent with previous studies [19, 20], cells overexpressing XIAP had a reduction in NLRP3 signaling activation, and inflammation-associated dyslipidemia induced by PA treatment was eliminated in these cells (Figure 10A and 10B). These results suggest that XIAP is involved in the regulation of the NLRP3 inflammasome-mediated inflammatory response in PA-treated hepatic cells.

**Figure 11:**
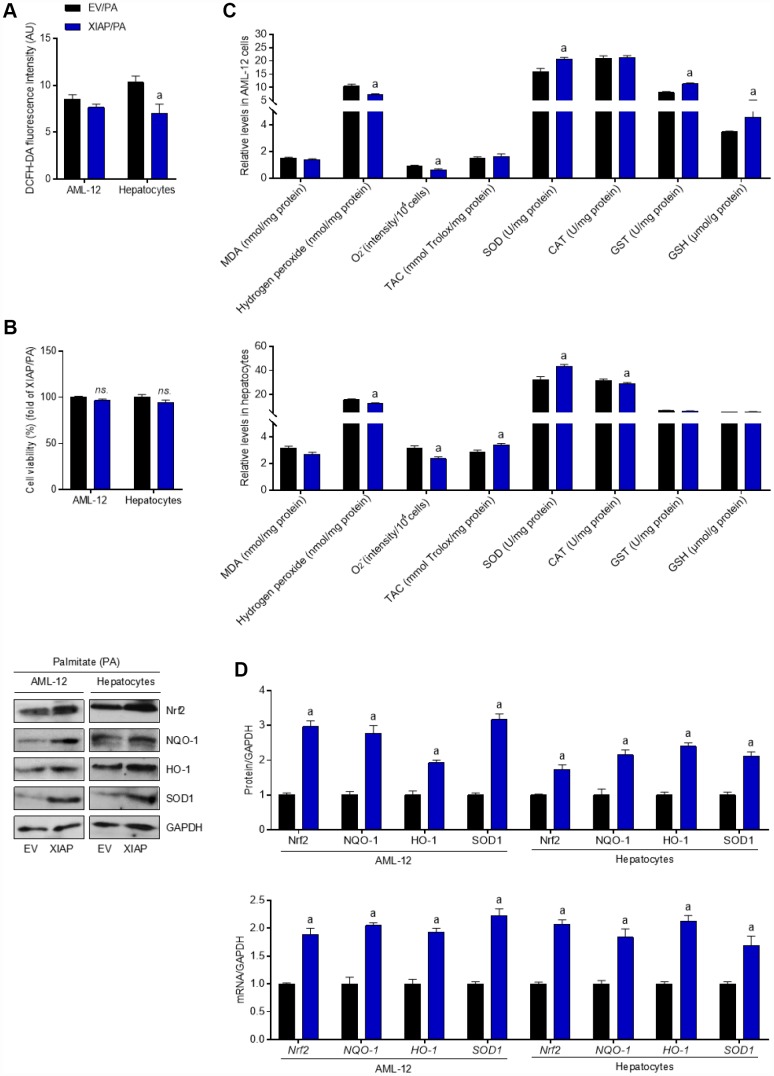
**Over expression of XIAP restrains PA-induced oxidative stress *in vitro*.** AML-12 and primary hepatocytes were transfected with pUNO1/XIAP plasmid for 24 h, followed by PA (250 μM) treatment for additional 24 h. Subsequently, further studies were detected. (**A** and **B**) Determination of cellular ROS production and cell viability. (**C**) Calculation of cellular MDA, H_2_O_2_, O_2_^-^ levels, TAC, SOD, CAT, GST and GSH activities. (**D**) Western blot and qPCR analysis showed the alteration of SOD1, NQO-1, HO-1 and Nrf-2 in cells treated as described. For all bar plots shown, data are expressed as the mean ± SEM. n = 8 per group. ^a^p < 0.05 vs. EV/PA.

Similar to the *in vivo* results, overexpression of XIAP resulted in both an increase in the generation of antioxidants and a decrease in the production of ROS without affecting cell viability (Figure 11A–11C), suggesting that XIAP regulates the PA-induced increase in oxidative stress injury in cells by regulating antioxidant production. Moreover, both Nrf2 activation and the expression of down-stream indicators were significantly increased in PA-treated cells compared to the EV group, indicating that XIAP plays an essential role in the regulation of Nrf2 activity in response to PA challenge (Figure 11D). Taken together, these results suggest that the expression of XIAP directly affects the oxidative stress response and antioxidant levels in hepatic cells under PA stress.

## DISCUSSION

NAFLD is a pathological process in which the liver develops steatosis and inflammation in response to excess fat accumulation, and it is considered the initial step toward hepatic fibrosis [41, 42]. Long-term consumption of a fat-rich diet can result in metabolic syndrome and systemic inflammatory response, leading to the development of hepatic insulin resistance, in which the ability of insulin to limit blood glucose production is abolished but its role in the promotion of lipid synthesis is preserved [3, 27, 28, 43]. The progression of insulin resistance leads to abnormal lipid accumulation in the liver [29, 30], suggesting that the mechanism underlying the development of NAFLD includes the elevation of free fatty acids in the systemic circulation. This reduction in both TG clearance and fatty acid β-oxidation results in an increase in *de novo* lipid synthesis, indicating that imbalances in lipid metabolism contribute to the progression of the disease [31–35]. Increases in the expression of CPT-1α and its upstream molecular, PPAR-α, in hepatocytes promote mitochondrial β-oxidation of fatty acids, rather than stimulate lipogenesis through the up-regulation of ACCα, FAS, or SCD1 expression [36, 37]. A series of membrane fatty acid transporters—including fatty acid transferases (differentiation clusters, CD36), the FATP family, and plasma membrane-related FABP—are known to play important roles in fatty acid uptake and synthesis [38–40]. Previous findings show that XIAP knockdown significantly reduced the clearance of circulating-lipids in mice fed with an HFD. XIAP knockdown also effectively up-regulated the expression of genes associated with fatty acid synthesis (ACCα, FAS, and SCD1) and intake (CD36, FATP1, and FABP1). Decreased XIAP expression also suppressed the expression of β-oxidation genes (CPT-1α and PPAR-α), which exacerbated lipid deposition in the liver. Therefore, XIAP is a potential therapeutic target for HFD-induced liver steatosis and related systemic metabolic diseases.

In this study, XIAP siRNA was used to establish an *in vivo* mouse model to determine whether XIAP acts as a negative regulator in HFD-induced NAFLD through the suppression of oxidative stress. Continuous consumption of an HFD promotes lipid accumulation in the liver by enhancing NLRP3 inflammasomes, promoting oxidative stress injury, and attenuating both Nrf2 activity and down-stream antioxidant generation via crosstalk with XIAP. Therefore, the effects of XIAP knockdown or overexpression on HFD-induced NAFLD in mice were investigated. Consistent with previous studies, it was found that XIAP knockdown promoted the NLRP3-mediated inflammatory response induced by long-term HFD in NAFLD. The data showed that free fatty acids enhanced NLRP3 activation and inflammatory cytokine generation, further promoting lipid accumulation in the liver. Notably, XIAP is associated with activation of the inflammatory response, which in turn negatively regulates the expression of NLRP3 inflammasomes and ultimately relieves the effects of NAFLD. However, XIAP knockdown resulted in the loss of this regulatory process in the face of systemic inflammatory stress, and the over-activation of NLRP3 exacerbated the progression of NAFLD. In contrast, overexpression of XIAP in hepatic cell lines resulted in the decrease of PA-induced NLRP3 activation, suggesting that XIAP mitigates lipid metabolism imbalance through the regulation of NLRP3-associated inflammatory responses *in vitro*. These results demonstrate that XIAP is an important anti-apoptotic regulatory factor that plays an important role in alleviating the effects of HFD-induced NAFLD.

Systemic inflammatory responses can effectively activate XIAP and NLRP3 activity. Excessive NLRP3 activation initiates the transcription and translation of downstream inflammatory cytokines, and a large number of inflammatory factors contribute to the progression of lipid metabolic disorders, accelerating the development of NAFLD. XIAP suppresses the assembly of NLRP3 inflammasomes by negatively regulating NLRP3 signaling, reducing the formation and release of inflammatory cytokines, and moderating hepatic steatosis. Previous studies have indicated that an HFD may repress XIAP expression partly due to the regulation of AKT expression. In this study, XIAP expression was shown to be downregulated in the liver of HFD mice. Decreases in XIAP expression may contribute to increases in both the NLRP3 signaling-associated inflammatory response and oxidative stress activation. Increases in NLRP3 activation may also recruit the XIAP/cIAP1/2 complex, further regulating the NLRP3-associated inflammatory response. However, overexpression of inflammatory cytokines derived from NLRP3 signaling can significantly suppress XIAP activation [19, 21]. This increased abundance of inflammatory cytokines could inhibit Nrf2 signaling, further diminishing the recruitment of XIAP. Thus, the reduction of XIAP levels in our mouse model may be due to the activation of both oxidative stress and the NLRP3-signaling pathway by free fatty acids in the systemic circulation.

Oxidative stress contributes to stimulant-triggered hepatic injury, possibly through up-regulating the production of ROS [44–46]. The balance between oxidative stress and oxidation resistance is essential for normal metabolic control. Overproduction of ROS and pro-oxidants, including superoxide radical, hydrogen peroxide, and MDA, can exacerbate oxidative stress injury by directly or indirectly inducing systemic inflammatory response, endoplasmic reticulum and mitochondrial damage, or cell apoptosis. Knockdown of XIAP expression in HFD-induced fatty livers increased oxidative stress levels and inflammatory infiltration, as well as triggered a significant reduction in Nrf2 activity and antioxidant production. This indicated that changes in XIAP levels were associated with the regulation of oxidative stress. PA-treated AML-12 cells and hepatocytes were used to model hepatic fat accumulation *in vitro*, and these cells exhibited higher ROS levels compared to non-treated controls. Moreover, PA-treated cells had significantly increased levels of MDA, superoxide radical, and hydrogen peroxide, as well as decreased levels of SOD, TAC, CAT, GST, and GSH. These results indicate that XIAP plays an important role in the regulation of oxidative stress in hepatocytes. Previous studies have linked increases in cellular oxidative stress to the development of NAFLD [44, 45]. The data presented here reveal that crosstalk between oxidative stress and systemic inflammatory response enhances hepatic lipid deposition and the progression of NAFLD. Knockdown of XIAP in the liver promoted the disturbance of hepatic lipid metabolism through the upregulation of genes associated with fatty acid synthesis and uptake. By contrast, overexpression of XIAP in hepatic cell lines reversed PA-induced oxidative stress injury and decreased the production of pro-oxidants, stress or inflammatory response through enhanced XIAP activity may also partly rescue Nrf2-mediated anti- oxidative stress and increase antioxidant generation, which could ultimately contribute to the recovery of metabolic balance.

**Figure 12:**
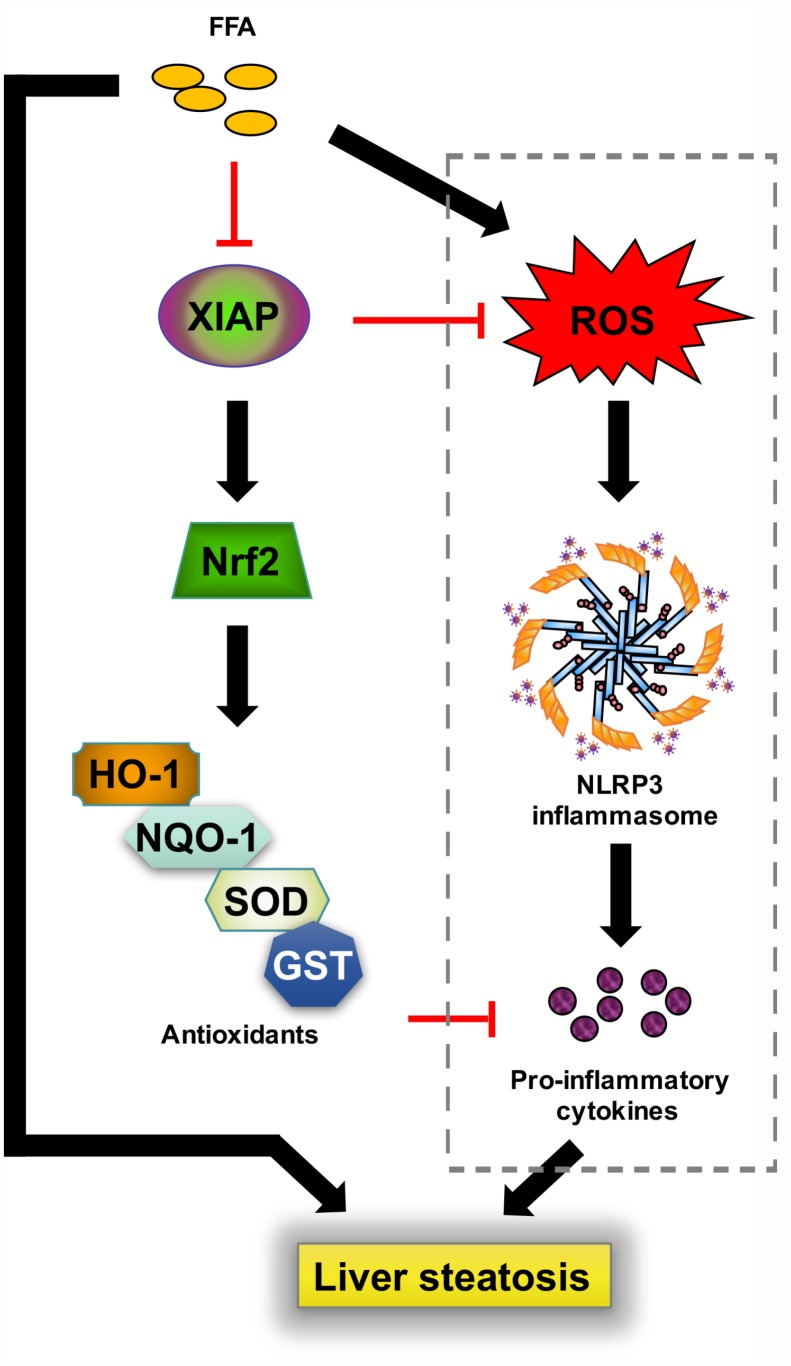
**Schematic diagram depicting the role of XIAP in HFD-induced steatohepatitis.** In response to a prolonged challenge with fat-rich diet ingestion, enhanced oxidative stress injury caused by circulatory free fatty acid promotes NLRP3 inflammasome mediated inflammation infiltration, followed by generation of down-stream pro-inflammatory cytokines, ultimately facilitates metabolic disorder associated lipid accumulation in liver. In contrast, activated XIAP and Nrf2 activity are associated with decreased oxidative stress. Circulatory free fatty acid may suppress XIAP expression, further reduce Nrf2 pathway activation. Up-regulation of XIAP may act as the upstream to inhibit ROS generation and induce Nrf2 and other antioxidants activity. In addition, elevated Nrf2 activity and down-stream anti-oxidants including HO-1, NQO-1, SOD or GST further inhibit inflammatory response, finally relieve lipid deposition in liver and hepatic inflammation.

The current work has characterized the protective role of XIAP in HFD-induced NAFLD by regulation of oxidative stress and NLRP3-mediated inflammation (Figure 12). XIAP knockdown promoted HFD-induced inflammatory infiltration by activating NLRP3 activity and pro-inflammatory cytokine generation, further accelerating hepatic lipid accumulation. In addition, oxidative stress injury induced by HFD consumption was enhanced by knocking down XIAP expression. Increased oxidative stress further exacerbated hepatic inflammatory responses, while impaired Nrf2 signaling and downstream antioxidant production resulted in the amelioration of hepatic steatosis and related complications. Given the multifactorial and multi-stage characteristics of NAFLD pathogenesis, none of the molecules under exploration for NAFLD treatment would be able to treat all patients. Therefore, further research aimed to identify new targets for the prevention and treatment of NAFLD is desperately needed.

The current study indicated that fat-rich diet-induced NAFLD exhibited lower XIAP and Nrf2 activity levels, then mediated an increase in NLRP3 inflammasomes, resulting in hepatic inflammation and dyslipidemia. Therefore, the activation of higher XIAP expression to handle NLRP3 inflammasome-related inflammatory responses by cross talking with the anti-oxidative stress system may be a novel therapeutic strategy.

## MATERIALS AND METHODS

### Study approval and ethics statement

All study protocols associating with animals were approved by the Institutional Animal Care and Use Committee in Guangzhou University of Chinese Medicine and Chongqing Key Laboratory of Medicinal Resources in the Three Gorges Reservoir Region, School of Biological and Chemical Engineering, Chongqing University of Education. The animals received humane care according to the criteria outlined in the Guide for the Care and Use of Laboratory Animals prepared by the National Academy of Sciences and published by the National Institutes of Health in 1996. The methods used in this work also were in accordance with the Regulations of Experimental Animal Administration issued by the Ministry of Science and Technology of the People's Republic of China (http://www.most.gov.cn).

### Animals and experiment design

Male mice (C57BL/6; wild type) at the age of 6-8 weeks, weighing 18–25 g, were purchased from the Beijing Vital River Laboratory Animal Technology Co., Ltd (Beijing, China). A total of 210 mice were used in this study. Prior to all experiments, the mice were allowed to adapt to the environment for 7 days. The mice were maintained in a constant temperature (controlled by GREE air conditioner), humidity and pathogen-free-controlled environment (25 °C ± 2 °C, 50% ± 3 %) with a standard 12 h light/12 h dark cycle, abundant food and water (pathogen-free) in their houses. After one-week acclimatization, mice with nonalcoholic fatty liver disease model were constructed by feeding with high fat diet (HFD) fodder (20% kcal protein, 60 kcal% fat and 20% kcal carbohydrate, Cat#: D12492;Research Diets, New Brunswick, NJ, USA) for 12 weeks until experimental mice were sacrificed for further study. In addition, mice that were fed a standard chow diet (SCD)(20% kcal protein, 10 kcal% fat and 70% kcal carbohydrate, Cat#: D12450H; Research Diets, New Brunswick, NJ, USA)as control group. To generate *in vivo* knockdown mice, XIAP siRNA *in vivo* transfection (ItsiXIAP) was performed by injecting with a XIAP siRNA (Generay Biotech Co.Ltd., Shanghai, China). A scrambled siRNA was used as a negative control (ItsiRNA). All mice were randomly divided into 4 groups: (1) ItsiRNA/SCD; (2) ItsiXIAP/SCD; (3) ItsiRNA/HFD and (4) ItsiXIAP/HFD. The XIAP siRNA or scrambled siRNA (50 ug) was diluted in 50 uL of Entranster^TM^-*in vivo* RNA transfection reagent (Engreen Biosystem Co., Ltd., Beijing, China) and 10% glucose(Cat#: G6172, Macklin Inc., Shanghai, China) mixture according to the manufacturer’s instructions. A volume of 50 uL siRNA or siXIAP mix was continuously subjected to mice by tail vein every 10 days for a total of 12 weeks according to the reagent’s instructions. The generation of *in vivo* knockdown mice preparation and protocol was performed in accordance with our previous work [46]. The body weight of all mice was recorded every 4 weeks. After SCD or HFD feeding for 12 weeks, blood was sampled from removing the eyeball for the plasma analysis. Subsequently, the liver tissues were carefully and quickly removed for weight measurement, and/or in part dissected for RT-PCR, western blot, immunohistochemical assay or other biochemical analysis.

### Biochemical analysis

For glucose tolerance test (GTT) and insulin tolerance tests (ITT) test, all mice associating with this regard were fasted for 8 h to confirm the correction of physiological response. For GTT test, mice were orally gavaged with glucose (2 g/kg body weight) (Cat#: D810588, Macklin Inc., Shanghai, China). Then, the concentration of blood glucose of tail venous blood at 0 min, 30 min, 60 min, 90 min and 120 min after glucose treatment was examined using commercial blood glucose test strips (ACCU-CHEK^®^, Roche Diabetes Care GmbH, Shanghai, China). For insulin resistance detection, mice were treated with an intraperitoneal injection of insulin (1 U/kg body weight, Sigma Aldrich). The GTT and ITT analysis protocol were used in accordance with our previous study [46]. Subsequently, blood samples were collected from the tail vein at 0 min, 30 min, 60 min, 90 min and 120 min post-injection for measurement of glucose levels. Serum AST (glutamic-oxalacetic transaminase), ALT (glutamic-pyruvic transaminase), AKP (alkaline phosphatase), serum insulin and TG (triglyceride), TC (total cholesterol) in liver or serum were next detected using commercially-available detection kits (Nanjing Jiancheng Bioengineering Institute, Nanjing, China). The serum endotoxin was also determined using mouse endotoxin, ET ELISA Kit (Nanjing SenBeiJia Biological Technology Co., Ltd., Nanjing, China). The glycosylated hemoglobin (HbA1c) of mice was further detected using commercial mouse HbA1c ELISA Kit (Nova Lifetech Inc.).

### Cells culture and treatment

Primary hepatocytes were isolated from the liver of 6-8-week-oldmale mice using the collagenase perfusion method as previously described [25, 26]. In brief, after the liver tissues were digested with a perfusion of collagenase type IV solution (Gibco™, Thermo Fisher Scientific, USA), the liver was excised, minced and filtered using a 70-μm cell strainer (BD Falcon, USA). The resulting solution was centrifuged to harvest hepatocytes. Then, the primary hepatocytes were separated and purified via Percoll solutions (Sigma Aldrich). Next, the purified hepatocytes were cultured in collagen-coated plates in DMEM (Gibco Corporation, Gaithersburg, MD, USA) supplemented with 10% fetal bovine serum (Gibco Corporation, Gaithersburg, MD, USA) and 1% penicillin-streptomycin(Gibco Corporation, Gaithersburg, MD, USA). The normal mice cultured AML-12 cells were purchased from ATCC (American Type Culture Collection), and then cultured at 37 °C, 5% CO_2_ in DMEM/F12 medium (Gibco Corporation, Gaithersburg, MD, USA) containing 10% fetal bovine serum, 1×10^5^ U/L streptomycin (Gibco Corporation, Gaithersburg, MD, USA). Then, confluent cultures were passaged by trypsinization (Hyclone) and maintained in 12-well, 24 well or 96-well plates (Corning, Shanghai, China) at a density of 2×10^5^/ml or 1×10^4^/ml for next 3 days. Subsequently, to calculate the inflammatory response and explore the activity of XIAP signaling, XIAP specific siRNA (5’-AATAGTGCCACGCAGTCTA-3’) and pUNO1 bearing the mouse XIAP plasmid (InvivoGen, HongKong, China), and corresponding negative control sequence or empty vector were subjected to cells using Lipofectamine 3000 Reagent (Invitrogen, Thermo Fisher Scientific, USA). For the effects detection of XIAP signaling, the cells were next treated with 250 μM palmitate (PA) (Sigma) for 24 h. Then, the cells were collected for the further study. The WST-8 assay [2-(2-Methoxy-4-nitrophenyl)-3-(4-nitrophenyl)-5-(2,4-disulfophenyl)-2 Htetrazolium Sodium Salt] was used to examine the 24 h siRNA and/or PA co-treated cells viability by using Cell Counting Kit 8 (CCK-8) (Solarbio, Beijing, China) according to the manufacturer instructions.

### Oxidative stress analysis *in vivo* and *in vitro*

Assay kits for detection of malondialdehyde (MDA) (Cat#: A003-1), superoxide dismutase (SOD) (Cat#: A001-3), catalase (CAT) (Cat#: A007-1-1), and glutathione (GSH) (Cat#: A005) were obtained from Nanjing Jiancheng Bioengineering Institute (Nanjing, China). Hydrogen peroxide (H_2_O_2_) (Cat#: S0038), O_2_^-^ (Cat#: S0060), inducible nitric oxide synthase (iNOS) (Cat#: S0025) and total antioxidant capacity (TAC)(Cat#: S0116) levels were tested using corresponding kits from commercially available kits (Beyotime, Nanjing, China). Xanthine oxidase (XO) (Cat#: K710, Biovision, San Francisco, USA) and glutathione S-transferase (GST) (Cat#: ABIN773241, Abnova) were also introduced into this regard to determine the corresponding levels of indicators. In addition, 2’,7’-Dichlorofluorescein diacetate (DCFH-DA) stain for ROS measurement reagents (Cat#: S0033) was purchased from Beyotime (Nanjing, China).

### Real-time quantitative PCR and immunoblotting assay

In this regard, TRIzol product (Cat#:15596026, Invitrogen, Thermo Fisher Scientific, USA) was used to harvest the total RNA samples from cells or liver tissues. Specifically, 1 μg of total RNA extraction was reverse transcribed using the M-MLV-RT system (Invitrogen^TM^, Shanghai, China). The program was performed at 42 °C for 1 h and terminated by deactivation of the enzyme at 70 °C for 10 min. PCR were conducted using SYBR Green (Bio-Rad) in BIO-RAD CFX96 touch q-PCR detection systems (Bio-Rad). Pre-denatured products for amplification was performed at 94 °C for 55s; followed by 45 cycles at 95 °C for 30s, 57 °C for 30s and 73 °C for 30s; followed by 95 °C for 10s, 65 °C for 45s, and 40 °C for 60s. In brief, fold induction values were calculated according the 2^-ΔΔCt^ expression, where ^Δ^Ct represents the differences in cycle threshold number between the target gene and GAPDH, and ^ΔΔ^Ct represents the relative change in the differences between control and treatment groups. The primer sequences for RT-PCR were produced by Sangon Biotech (Shanghai) Co., Ltd., and indicated in Table 2. For immunoblotting analysis, cells or tissues were homogenized into 10% (wt/vol) hypotonic lysis buffer (pH 8.0, 1mM EDTA, 50 μg/ml aprotinin, 25mM Tris-HCl, 5 μg/ml leupeptin, 1mM Pefabloc SC, 4mM benzamidine, 5 μg/ml soybean trypsin inhibitor) to yield a homogenate. Next, the final liquid supernatants were harvested by centrifugation at 13500 rpm for 30 min. Protein concentration was measured using Pierce^TM^ Rapid Gold BCA Protein Assay Kit (Thermo, USA) with bovine serum albumin as a standard. The total protein extraction will be performed for western blot analysis. Equal amounts of total protein of tissues were subjected to 10% or 12% SDS-PAGE and then transferred to a 0.45 μM PVDF membrane (Millipore Company, USA) followed by immunoblotting using the primary antibodies. The primary antibodies used in this study included anti-GAPDH (K106389P, dilution 1:5000, Solarbio, China), anti-XIAP (ab21278, dilution 1:1500, Abcam), anti-ACC (3676, dilution 1:1000, Cell Signaling Technology), anti-p-ACC (11818, dilution 1:1000, Cell Signaling Technology), anti-Fas (ab82419, dilution 1:1250, Abcam), anti-SCD1 (2794, dilution 1:1500, Cell Signaling Technology), anti-Caspase-8 (ab25901, dilution 1:1000, Abcam), anti-NLRP3 (ab214185, dilution 1:1000, Abcam), anti-ASC (sc-365611, dilution 1:300, Santa Cruz Biotechnology), anti-Caspase-1 (ab1872, dilution 1:1000, Abcam), anti-Nrf2 (ab62352, dilution 1:1000, Abcam), anti-HO-1 (ab13248, dilution 1:1000, Abcam), anti-SOD1 (ab13498, dilution 1:1000, Abcam), anti-NQO-1 (ab28947, dilution 1:1000, Abcam), anti-IL-18 (ab71495, dilution 1:1000, Abcam) and anti-IL-1β (#31202, dilution 1:1000, Cell Signaling Technology). Then, the membranes were blocked with 5% skim milk (Difco^TM^ Skim Milk, BD, USA)in 1×TBS buffer (Cat#: T1080, Solarbio, Beijing, China) containing 0.1% Tween-20 (1247ML100, BioFROXX, Germany) (TBST) for 1 h and incubated with the primary antibodies overnight (4°C). Membranes were rinsed in TBST and incubated with HRP-conjugated anti-rabbit or anti-mouse secondary antibodies (Abcam) for 2 h at room temperature. Bands were visualized by Pierce^TM^ ECL Western Blotting Substrate (Thermo Scientific, China) and exposed to Kodak (Eastman Kodak Company, USA) X-ray film. Corresponding protein expression will be determined as grey value (Version 1.4.2b, Mac OS X, ImageJ, National Institutes of Health, USA) and standardized to housekeeping genes (GAPDH) and expressed as a fold of control.

**Table 2:** The primer sequences for the targeted mouse genes.

**Primers**	**Forward Sequence (5’-3’)**	**Reverse Sequence (5’-3’)**
XIAP	GGCCATCTGAGACACATGCAG	GCATTCACTAGATCTGCAACC
CD36	AAATAAACCTCCTTGGCCTGA	GCAACAAACATCACCACACC
FATP1	GATGTGCTCTACGACTGTCTG	CAGCCCATAGATGAGACACTG
FABP1	GCAGAGCCAGGAGAACTTTG	GGGTCCATAGGTGATGGTGAG
CPT-1α	TCTTGCAGTCGACTCACCTT	TCCACAGGACACATAGTCAGG
PPARα	CAAGGCCTCAGGGTACCACT	TTGCAGCTCCGATCACACTT
Nrf-2	TGAAGTTCGCATTTTGATGGC	CTTTGGTCCTGGCATCTCTAC
HO-1	TGAACACTCTGGAGATGACA	GGACTCTGGTCTTTGTGTTC
NQO-1	GGTTTACAGCATTGGCCACACT	AACAGGCTGCTTGGAGCAAA
NLRP3	CCAGGGCTCTGTTCATTG	CCTTGGCTTTCACTTCG
ASC	CTCACCGCTAACGTGCTG	CCGGTGCTGGTCTATAAAGTG
Caspase-1	TGGTCTTGTGACTTGGAGGA	TGGCTTCTTATTGGCACGAT
Caspase-8	TGTTGGAGGAAAGCAATCTG	CCTGGTGTCTGAAGTTCCCT
SOD1	CTTCTCGTCTTGCTCTCTCTGG	TCCTGTAAATTTGTCCTGACAACAC
GCLM	CACAATGACCCGAAAGAACTG	AGACTTGATGATTCCCCTGCT
ACCα	GGCCAGTGCTATGCTGAGAT	AGGGTCAAGTGCTGCTCCA
FAS	CTGCGGAAACTTCAGGAAATG	GGTTCGGAATGCTATCCAGG
SCD1	CTTCTTGCGATACACTCTGG	TGAATGTTCTTGTCGTAGGG
GAPDH	GGTGAAGGTCGGTGTGAACG	CCCGTAGGGCGATTACAGTC

### Histological assay

Fixed white adipose tissue (WAT) and liver sections were embedded in paraffin; sections were stained to visualize the pattern of lipid accumulation and the inflammatory status of tissues. Four fields from each WAT section were analyzed to obtain the mean cell area [46, 47]. Briefly, the mice liver and white adipose tissues were fixed with 4% paraformaldehyde (Cat#: P1110, Solarbio, China), embedded in paraffin (Cat#: P100928, Aladdin, Shanghai, China), and sectioned transversely. Thin sections were stained with hematoxylin and eosin (H&E) (Hematoxylin and Eosin Staining Kit, Yeasen, Shanghai, China) and sirius red staining according to a standard histopathological processes. All sections were detected by 3 histologists without knowledge of the treatment procedure. The criteria regarding the evaluation of NAFLD associated histopathological changes were followed to the Kleiner or the Brunt criteria [48]. To visualize lipids accumulation, liver tissues were frozen in Tissue-Tek OCT (Tissue-Tek, Sakura Finetek, USA) and sections were then stained with Oil Red O Stain Kit (Nanjing Jiancheng Bioengineering Institute, Nanjing, China) for 10 min. After being rinsed with 60% isopropyl alcohol (Cat#: I811925, AR, Macklin Inc., Shanghai, China), the tissue sections were re-stained with hematoxylin. For immunohistochemistry, embedded sections were deparaffinized before administration with primary antibodies at 4 °C overnight. The tissues were subjected to immunohistochemical (IHC) staining for the measurement of XIAP (ab21278, dilution 1:300, Abcam), Caspase-8 (ab25901, dilution 1:250, Abcam), Nrf2 (ab62352, dilution 1:250, Abcam), HO-1 (ab13248, dilution 1:250, Abcam) or SOD1 (ab13498, dilution 1:250, Abcam) expression. All the histological procedure was performed in accordance with the standard procedures as indicated in reagent specifications. All the images were captured using optical microscope (Olympus, Japan). Four fields from each WAT section were analyzed to obtain the mean cell area. The evaluation of immunohistochemical (IHC) and adipocyte size were determined using Image J software derived from National Institutes of Health (NIH), USA. The number of positive cells per high power field (HPF) for quantification of IHC staining was counted using Image J. Antibody staining was assessed and scored using the “Quick Score method” [49]. Briefly, the proportion of positive cells was estimated and scored on a scale of 1 to 6; 0–4% = 1, 5–19% = 2, 20–39% =3, 40–59% = 4, 60–79% = 5, and 80–100% = 6.

### Statistical analysis

Results were expressed as mean ± standard error of the mean (SEM) from three independent experiments. Comparisons between groups were analyzed by one-way ANOVA followed by Dunnett's multiple comparisons test. GraphPad Prism Software (version 7.0 for Mac OS X Snow Leopard; Graph Pad Software, Inc., San Diego, CA) was used for the analysis. A p-value less than 0.05 will be considered significant.

## Supplementary Material

Supplementary Figure 1
