# Supplementary material for: Effects of XIAP on high fat diet-induced hepatic steatosis: a mechanism involving NLRP3 inflammasome and oxidative stress
Source: Aging (Albany NY). 2019 Dec 16;11(24):12177–201. doi: 10.18632/aging.102559 (PMC6949096; doi:10.18632/aging.102559)
Supplement: Supplementary Figure 1 [file aging-11-102559-s001..pdf]

SUPPLEMENTARY FIGURE

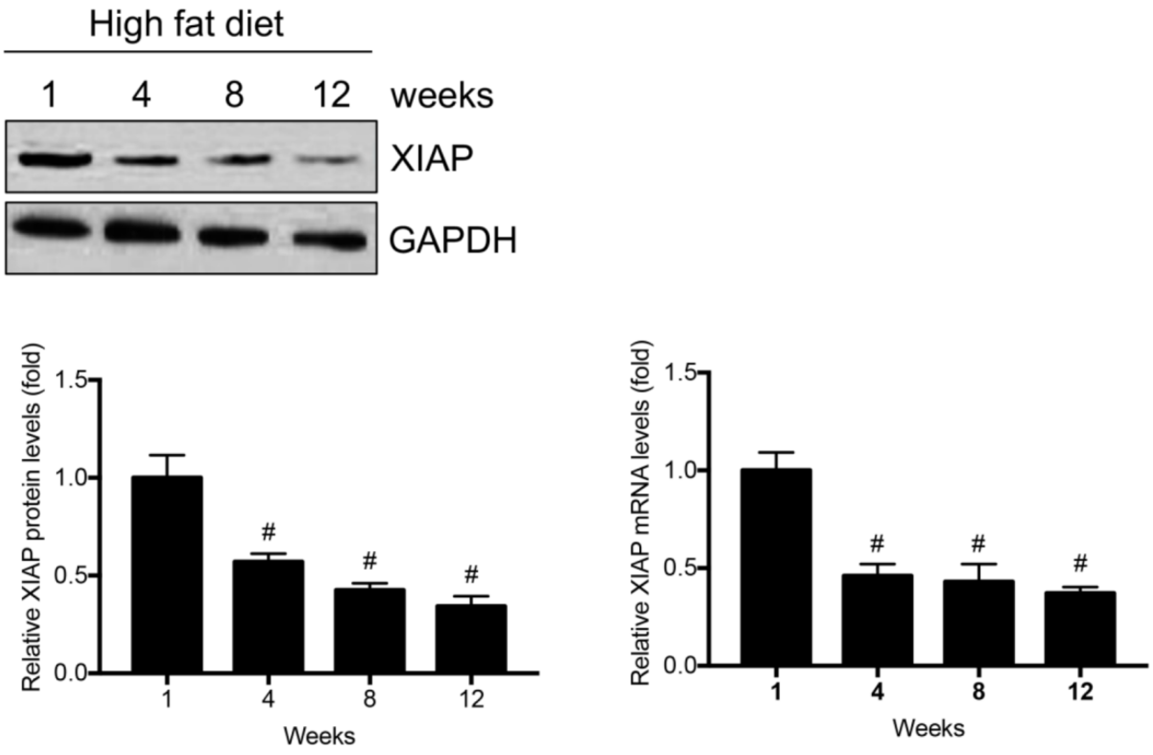

Supplementary Figure 1. The efficacy of XIAP siRNA *in vivo* in high fat induced NAFLD. XIAP gene or protein levels were detected in the liver tissues in 1st, 4th, 8th, and 12th week to determine the efficacy of XIAP siRNA *in vivo*. For all bar plots shown, data are expressed as the mean ± SEM. n = 8 per group. #$p < 0.05$ vs. 1st week.
